# Prognostic implications of PD-L1 expression in gastric cancer: systematic review and meta-analysis of studies published between 2018 and 2025

**DOI:** 10.1186/s12885-026-15880-z

**Published:** 2026-03-23

**Authors:** Gulnaz Yessultanova, Zhanat Komekbay, Indira Karibayeva, Anar Tulyayeva, Nurgul Kereyeva, Aigul Zhumasheva, Lunara Ishimova

**Affiliations:** 1 Department of Histology, West Kazakhstan Marat Ospanov Medical University, Aktobe, 030012 Kazakhstan; 2 Department of Health Policy and Community Health, Jiann-Ping Hsu College of Public Health, Georgia Southern University, Statesboro, GA 30460 USA; 3 Department of Oncology, West Kazakhstan Marat Ospanov Medical University, Aktobe, 030012 Kazakhstan; 4 Department of Pathomorphology, Medical Centre of West Kazakhstan Marat Ospanov Medical University, Aktobe, Kazakhstan; 5 Department of Public Health and Healthcare, West Kazakhstan Marat Ospanov Medical University, Aktobe, 030012 Kazakhstan

**Keywords:** Gastric cancer, PD-L1, Prognostic, Overall survival

## Abstract

**Background:**

Programmed death-ligand 1 (PD-L1) is widely used as a predictive biomarker for response to immune checkpoint inhibitors in gastric cancer (GC); however, its independent prognostic value for overall survival remains controversial. Differences in PD-L1 detection methods, scoring systems, and tumor immune microenvironment characteristics may contribute to inconsistent findings across studies. Therefore, this systematic review and meta-analysis aimed to evaluate the association between PD-L1 expression and overall survival in patients with GC.

**Methods:**

A systematic search of PubMed, Scopus, MEDLINE, and Web of Science was conducted to identify eligible studies published between January 2018 and December 2025. Cohort studies reporting hazard ratios (HRs) for overall survival in relation to PD-L1 expression assessed by immunohistochemistry were included. A random-effects meta-analysis was performed to estimate pooled effect sizes. Heterogeneity was assessed using the I^2^ statistic, and subgroup analyses were conducted according to PD-L1 assessment methods. Meta-regression analyses were performed to explore potential sources of heterogeneity. Study quality was evaluated using the Newcastle–Ottawa Scale, and the certainty of evidence was assessed using the GRADE approach.

**Results:**

Seven retrospective cohort studies comprising 1,993 patients with GC were included in the analysis. PD-L1 expression was significantly associated with overall survival (HR = 1.70, 95% CI: 1.13–2.55), although the direction of the prognostic effect varied across studies (I^2^ = 69.1%). Studies using the combined positive score (CPS ≥ 1) demonstrated more consistent prognostic associations compared with those employing semi-quantitative scoring systems. Meta-regression analyses did not identify significant modifying effects of publication year, mean age, or sex distribution on the pooled estimates. The overall certainty of evidence was rated as moderate.

**Conclusion:**

PD-L1 expression is associated with overall survival in GC; however, its prognostic implications appear to be context-dependent and influenced by methodological differences in PD-L1 assessment and tumor immune microenvironment characteristics. Standardization of PD-L1 evaluation methods and integration with molecular tumor subtypes may improve its prognostic utility in GC.

**Trial registration:**

https://www.crd.york.ac.uk/PROSPERO/view/CRD420251249939, identifier CRD420251249939.

## Introduction

Gastric cancer (GC) is among the five most common malignant cancers worldwide and ranks as the second leading cause of cancer-related death, with adenocarcinomas accounting for more than 95% of cases [1–4]. Despite advances in conventional treatments, including surgery, radiotherapy, and chemotherapy, improvements in patient outcomes have been limited. According to the Global Burden of Disease 2021 Study, both the incidence and mortality of GC increased between 1990 and 2021, with more than 1.23 million new cases, approximately 955,000 deaths, and an estimated 22.8 million disability-adjusted life years (DALYs) reported globally [5]. Consequently, there is increasing interest in immunotherapy approaches targeting immune checkpoints in GC. Monoclonal antibodies directed against programmed cell death-1 (PD-1) and programmed death-ligand 1 (PD-L1) have emerged as promising treatments due to their notable efficacy and favorable safety profile [6, 7].

PD-L1 expression has been extensively studied as a potential biomarker for response to PD-1/PD-L1 inhibitors; nevertheless, its prognostic and predictive value in GC remains controversial. Several studies and meta-analyses have reported that PD-L1 overexpression is associated with advanced tumor stage, lymph node metastasis, and poorer overall survival, suggesting a negative prognostic role in GC [8, 9]. The therapeutic landscape of cancer treatment has evolved substantially over the past decades. Conventional treatment strategies such as surgery, chemotherapy, and radiotherapy historically represented the cornerstone of GC management; however, their impact on long-term survival has remained limited, particularly in advanced disease. In recent years, the development of immunotherapy, particularly immune checkpoint inhibitors targeting PD-1 and its ligand PD-L1, has transformed the treatment paradigm of several malignancies, including GC. These therapies restore anti-tumor immune responses by blocking inhibitory signaling pathways that suppress T-cell activation, thereby enabling immune-mediated tumor elimination. As a result, PD-L1 expression has emerged not only as a predictive biomarker for response to immune checkpoint inhibitors but also as a potential prognostic indicator reflecting the interaction between tumor cells and the host immune system.

In contrast, other investigations have demonstrated that PD-L1 positivity correlates with improved survival outcomes or favorable immune-related features, particularly in tumors with microsatellite instability or Epstein–Barr virus positivity, indicating a context-dependent prognostic significance [10, 11].

The interpretation of PD-L1 as a prognostic biomarker in GC remains challenging due to methodological heterogeneity across studies. In particular, differences in PD-L1 scoring approaches used across studies, including the CPS and semi-quantitative scoring systems, may substantially influence the reported prognostic associations. Differences in scoring methods and cut-off thresholds may therefore contribute to inconsistent findings regarding the prognostic significance of PD-L1 expression in GC [12].

A previous systematic review and meta-analysis by Gu et al. (2017) comprehensively evaluated the association between PD-L1 expression and prognosis in GC, synthesizing evidence published up to 2017 and reporting that PD-L1 overexpression was associated with unfavorable survival outcomes [13].

Recent advances in immunotherapy have significantly changed the therapeutic landscape of GC, particularly following the clinical implementation of immune checkpoint inhibitors targeting the PD-1/PD-L1 signaling pathway. These developments have substantially expanded the clinical and translational research on PD-L1 as both a predictive and prognostic biomarker in GC [14].

Since then, a substantial number of new observational studies and clinical trials have been published, alongside the widespread clinical implementation of immunotherapy in GC, warranting an updated and more comprehensive synthesis of the available evidence.

Therefore, this systematic review and meta-analysis aim to synthesize the current evidence on the association between PD-L1 expression in tumor tissue and overall survival in adult patients with histologically confirmed GC. The primary objective is to evaluate the prognostic significance of PD-L1 expression for overall survival in PD-L1 positive patients.

## Materials and methods

The study protocol is registered with the National Institute for Health Research’s PROSPERO International Prospective Register of Systematic Reviews [15] (ID: CRD420251249939).

### Search strategy

An initial search of the PROSPERO database to identify registrations of comparable studies revealed no previously registered systematic reviews or meta-analyses assessing the prognostic significance of PD-L1 expression in GC for the period 2018–2025. Given that the objective of the present study was to evaluate the association between PD-L1 expression in tumor tissue and overall survival in adult patients with histologically confirmed GC, the authors proceeded with registering the current study protocol in the PROSPERO database. Following this, a systematic search was conducted across four databases: PubMed, Scopus, MEDLINE, and Web of Science. The search commenced on 1 January 2018 and concluded on 11 December 2025. The search strategy utilized the keywords: “PD-L1” AND “gastric cancer” AND “prognosis”. Restrictions were applied to include studies published between 2018 and 2025; results were limited to English-language publications and studies conducted on human patients. Where applicable, filters were applied to include only original research articles and exclude other publication types, such as reviews, editorials, or conference abstracts.

### Eligibility criteria

The types of studies to be included were determined using the PECOS framework for eligibility criteria. The inclusion criteria were as follows: 1. Adult patients with histologically confirmed GC who underwent surgical treatment (total or partial gastrectomy). 2. Studies reporting PD-L1 expression in tumor tissue assessed by immunohistochemistry (IHC) using combined positive score (CPS) or semi-quantitative scoring methods. 3. Outcome: Hazard Ratio (HR) for overall survival in PD-L1 positive GC patients, calculated using Cox proportional hazards regression analysis. 3. Retrospective or prospective cohort studies published in English between 2018 and 2025. The exclusion criteria were as follows: 1. Patients who received radiotherapy or chemotherapy prior to PD-L1 assessment. 2. Studies assessing PD-L1 at mRNA, genomic, or transcriptomic levels, or in non-tumor samples (peripheral blood, serum, or immune cells). 3. overall survival presented in months or as the percentage of patients surviving over a specified period of time. 4. Reviews, systematic reviews, meta-analyses, editorials, letters, conference abstracts, case reports, or duplicate publications. 5. Studies published before 2018 or in languages other than English.

Studies including patients who received postoperative adjuvant chemotherapy or radiotherapy were not excluded, provided that PD-L1 expression was assessed in tumor tissue obtained at the time of surgery and that no systemic treatment had been administered prior to PD-L1 evaluation.

### Selection of studies and data extraction

The literature review and synthesis were conducted in accordance with the Preferred Reporting Items for Systematic Reviews and Meta-Analyses (PRISMA) guidelines [16]. Two authors (Zh.K and I.K) independently screened the titles and abstracts of the search results, following duplicate removal, to assess their relevance. Full texts of studies that met the initial criteria were retrieved and evaluated against the pre-defined inclusion and exclusion criteria. Data extracted from eligible studies included: authors, year of publication, country where the study was conducted, total number of patients, mean age, the proportion of male participants, tumor stage, the number of PD-L1–positive cases, PD-L1 expression assessment method, PD-L1 positivity definition, overall survival HR with 95% confidence interval (CI), and study design. Two authors (Zh.K and I.K) independently performed data extraction from the selected studies. Any discrepancies in data extraction were resolved through consultation with a third author (G.Y.) to ensure consensus among all three authors responsible for study selection and data extraction.

### Risk of bias (Quality) assessment

The studies included in this review were evaluated for risk of bias using the Newcastle–Ottawa Scale (NOS) for cohort studies, as recommended by the Cochrane Non-Randomized Studies Methods Working Group [17]. The NOS assesses studies across three domains: selection of study groups (0–4 points), comparability of groups (0–2 points), and outcome assessment (0–3 points), with a total possible score of 0–9; higher scores indicate better methodological quality. Studies scoring 0–3 points were considered to be of low methodological quality, those scoring 4–6 points were considered of moderate quality, and studies scoring 7–9 points were considered of high quality. Quality assessments were performed independently by two authors (Zh.K and I.K), with disagreements resolved through discussion with a third author (G.Y.). Overall, the included studies demonstrated moderate to high methodological quality, with evaluation scores ranging from 5 to 8, which supports the validity of the subsequent quantitative synthesis (Table 1).

**Table 1:** Newcastle–Ottawa risk of bias (quality) assessment results

	***First author***	***Year***	***Country***	Journal	Selection (0–4)	Comparability (0–2)	Outcome (0–3)	Total (0–9)	Quality
1	Wei [18]	2018	China	Translational Cancer Research	2	1	2	5	Moderate
2	Kim [19]	2020	Japan	Gastric Cancer	2	1	2	5	Moderate
3	Yamashita [20]	2020	Japan	Gastric Cancer	2	2	3	7	High
4	Liu [21]	2020	Korea	Pathology – Research and Practice	2	2	2	6	High
5	Lian [22]	2022	China	Digestive and Liver Disease	2	1	2	5	Moderate
6	Khalek [23]	2022	Egypt	Immunopathology & Pharmacology	2	2	3	7	High
7	Li [24]	2025	China	Cancer Medicine	3	2	3	8	High

### Statistical strategy for data synthesis

A random-effects meta-analysis was conducted in R version 4.5.1 using the meta and metafor packages within RStudio version 2023.06.1 + 524. HRs for overall survival in PD-L1 positive patients with GC were log-transformed and pooled using the DerSimonian and Laird method. Subgroup analyses were performed based on the method of PD-L1 assessment, specifically comparing CPS and semi-quantitative scoring approaches. To explore potential sources of heterogeneity, meta-regression analyses were conducted with publication year as a covariate. Sensitivity analyses included influence analysis to assess the robustness of the results. Publication bias was evaluated using visual inspection of funnel plots and Egger’s regression test. Sensitivity analyses were performed using a leave-one-out approach, in which each study was sequentially removed from the meta-analysis to assess its influence on the pooled effect estimate. This procedure allowed the robustness of the overall results to be evaluated and helped identify potentially influential studies contributing to heterogeneity.

### Certainty of evidence evaluation

To assess the certainty of evidence regarding the pooled HR for overall survival in PD-L1 positive patients with GC, we applied the GRADE (Grading of Recommendations Assessment, Development, and Evaluation) methodology, in accordance with the guidance outlined in the Cochrane Handbook for Systematic Reviews of Interventions [25]. The evaluation was carried out in Rstudio version 2023.06.1 + 524, using the standard GRADE criteria—risk of bias, inconsistency, indirectness, imprecision, and publication bias—to determine the overall certainty of evidence [26]. Based on these domains, the strength of the body of evidence could be rated as high, moderate, low, or very low. This systematic and transparent approach ensured a comprehensive appraisal of the confidence in the pooled estimate derived from the meta-analysis.

## Results

### Included study characteristics

The initial search of four databases (Web of Science, Scopus, PubMed, and MEDLINE) yielded a total of 2,531 articles**.** After removal of 1,099 duplicate records**,** 1,432 unique articles remained for title and abstract screening. During this stage, 1,373 articles were excluded as they did not meet the predefined eligibility criteria, leaving 58 articles for full-text review. Upon full-text assessment, 51 studies were further excluded for the following reasons: in 25 studies, the reported outcomes were not related to overall survival (OS); 11 studies did not report hazard ratios (HRs) for overall survival; in 9 studies, PD-L1 expression was assessed using methods other than those prespecified. The 3 studies included patients treated with chemotherapy [27–29], 2 reported only univariable analyses [30, 31], and 1 was restricted to advanced GC [32].

**Fig. 1:**
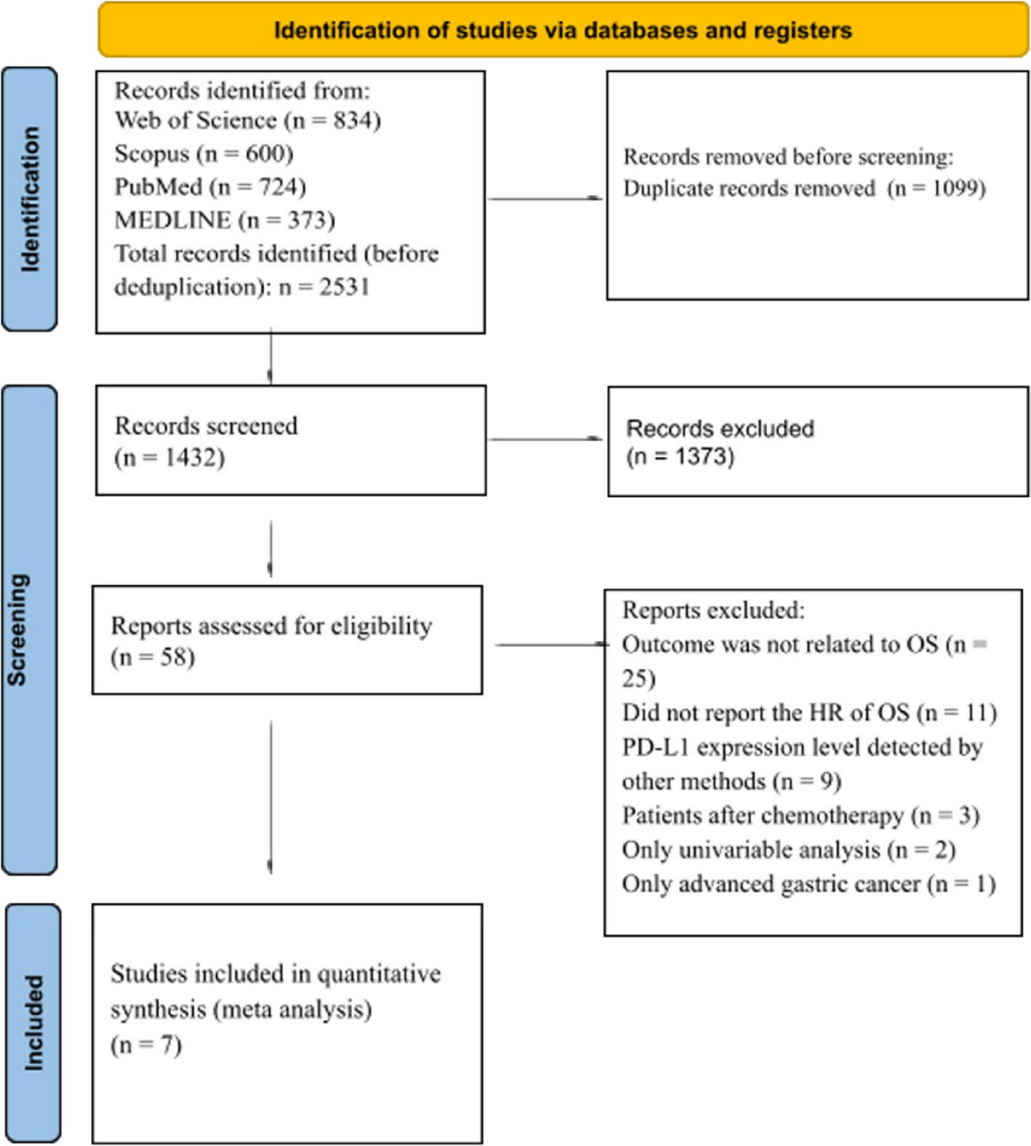
PRISMA flow diagram of study selection process [Page et al., 16]

As a result, seven studies fulfilled all inclusion criteria and were included in the quantitative synthesis (meta-analysis). A PRISMA flow diagram detailing the study selection process is presented in Fig. 1 [16].

The included studies were published between 2018 and 2025 and were conducted in East Asia and North Africa, including China (*n* = 3), Japan (*n* = 2), South Korea (*n* = 1), and Egypt (*n* = 1). All seven studies employed a retrospective study design. Collectively, the studies included a total of 1993 patients with GC, with individual sample sizes ranging from 75 to 1,007 patients.

The mean or median age of participants, where reported, ranged from approximately 60 to 69 years. The proportion of male patients varied across studies, ranging from 53.2% to 84%. Tumor stages included stage I–IV in most studies, while two studies were restricted to stage I–III disease.

PD-L1 expression was assessed using either semi-quantitative immunohistochemical scoring or the combined positive score. A CPS cut-off of ≥ 1 was applied in four studies. The proportion of PD-L1–positive tumors ranged from 29.1% to 71.7% across the included studies.

A detailed summary of study characteristics, including patient demographics, tumor stage, PD-L1 expression status, and PD-L1 assessment methods, is presented in Table 2.

**Table 2:** Description of the included studies to the meta-analysis

	***Author, year***	***Country***	***Total patients assessed***	***Mean age***	***Male (%)***	***Stage***	***PD-L1 positive (%)***	***HR (95% CI)***	***Assessment***
1	Wei [18]	China	86	65.6	67.4	I-IV	29.1%	2.351 (1.128—4.904)	Semi-quantItative
2	Kim [19]	Japan	286	60.8	66.1	I-IV	67.3%	0.229 (0.082—0.642)	Semi-quantItative
3	Yamashita [20]	Japan	137	68.9	72.3	I-III	71.7%	2.27 (1.27—4.37)	CPS ≥ 1
4	Liu [21]	Korea	300	64	66.3	I-IV	59.3%	1.793 (1.090—2.952)	Semi-quantitative
5	Lian [22]	China	75	62.17	84	I-IV	57.3%	1.87 (0.74—4.79)	CPS ≥ 1
6	Khalek [23]	Egypt	102	61.5	60.8	I-III	43.1%	7.502 (1.469—38.310)	CPS ≥ 1
7	Li [24]	China	1007	62.15	63.2	I-III	52.1%	1.759 (1.630—1.914)	CPS ≥ 1

**Fig. 2:**
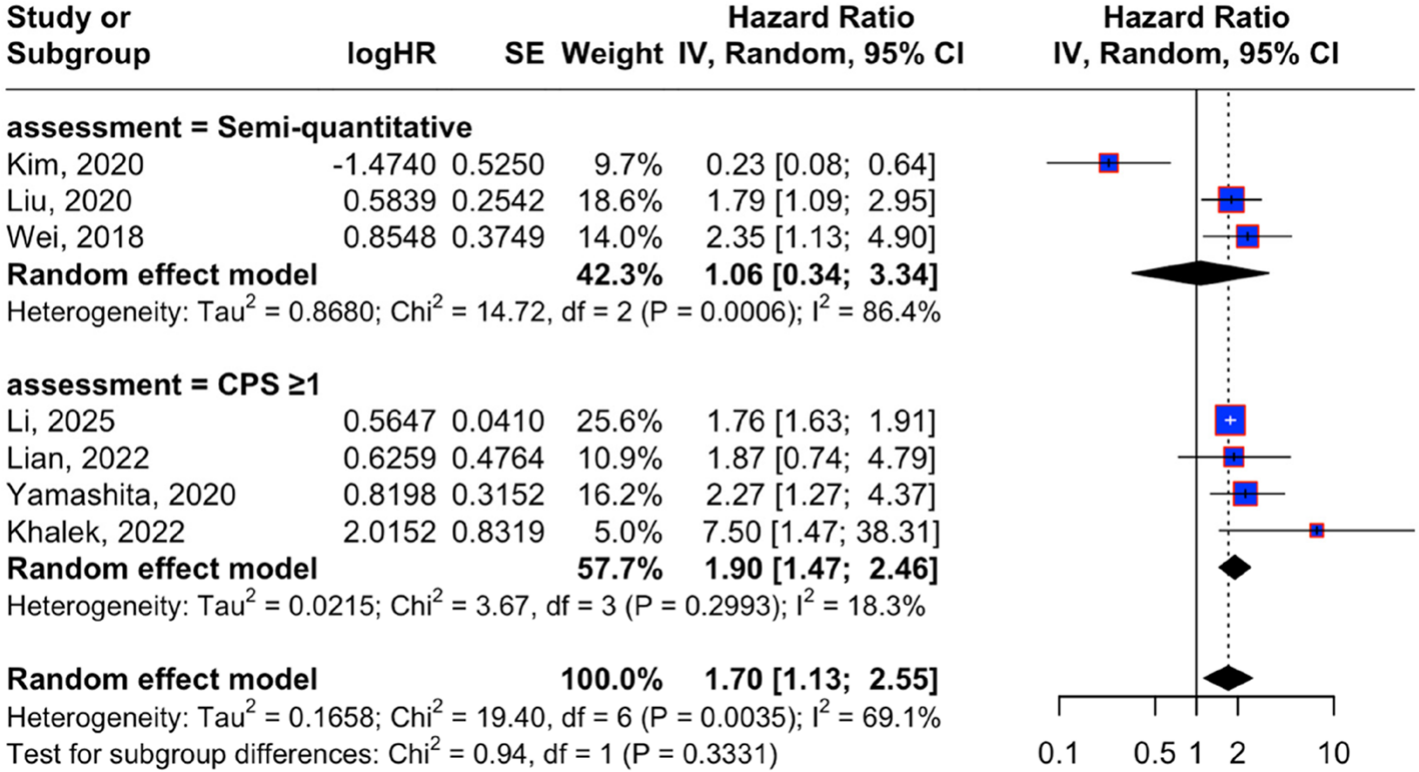
Forest plot of the association between PD-L1 expression and overall survival in gastric cancer

A total of seven studies included in the meta-analysis evaluated the association between PD-L1 expression and overall survival in patients with GC. The forest plot demonstrates that two of the three studies in the semi-quantitative subgroup reported an association between PD-L1 expression and improved overall survival, whereas studies in the CPS ≥ 1 subgroup showed more consistent prognostic associations, although the direction of the effect varied across individual studies. The overall pooled analysis using a random-effects model demonstrated that PD-L1 expression was significantly associated with overall survival in GC (HR = 1.70, 95% CI: 1.13–2.55), with moderately high heterogeneity (I^2^ = 69.1%, *p* = 0.0035), as presented in Fig. 2.

**Fig. 3:**
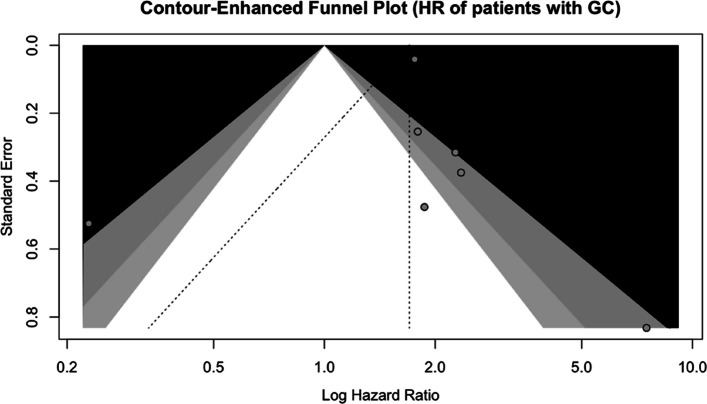
Funnel plot assessing publication bias in the association between PD-L1 expression and overall survival in gastric cancer

The funnel plot assessing potential publication bias among the included studies is presented in Fig. 3. Visual inspection of the funnel plot showed a largely symmetrical distribution of studies around the pooled effect size, suggesting the absence of substantial publication bias. This finding was further supported by non-significant Egger’s regression test (*p-value* = 0.81), as presented in Fig. 3.

**Fig. 4:**
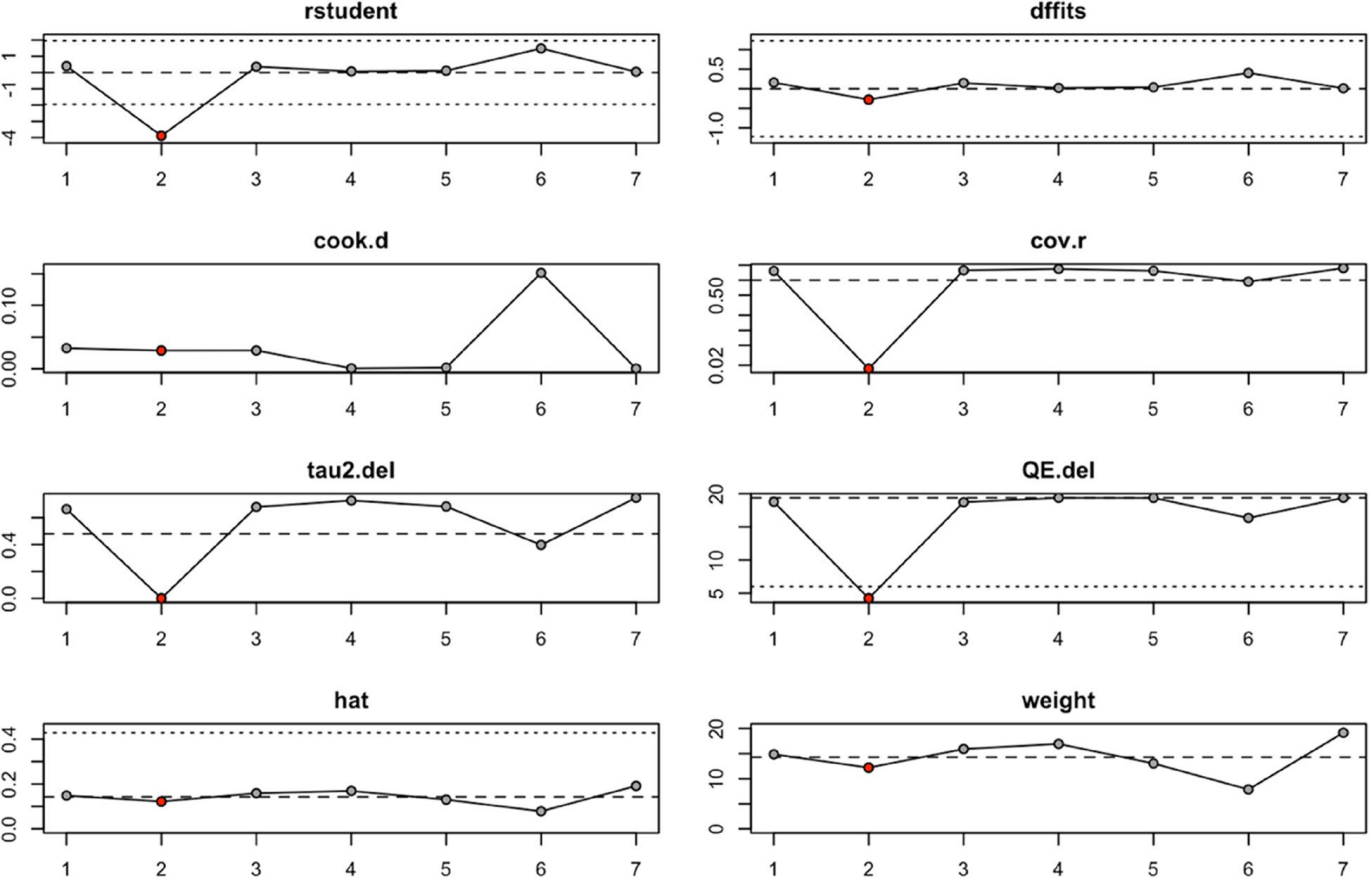
Influence analysis of individual studies on pooled overall survival hazard ratio in gastric cancer patients

An influence analysis was conducted to identify studies with the greatest impact on the pooled effect estimate, and the results are presented in Fig. 4. The analysis indicated that the pooled HR for overall survival was most influenced by the study of Kim et al., [19], as demonstrated in the influence plot.

**Fig. 5:**
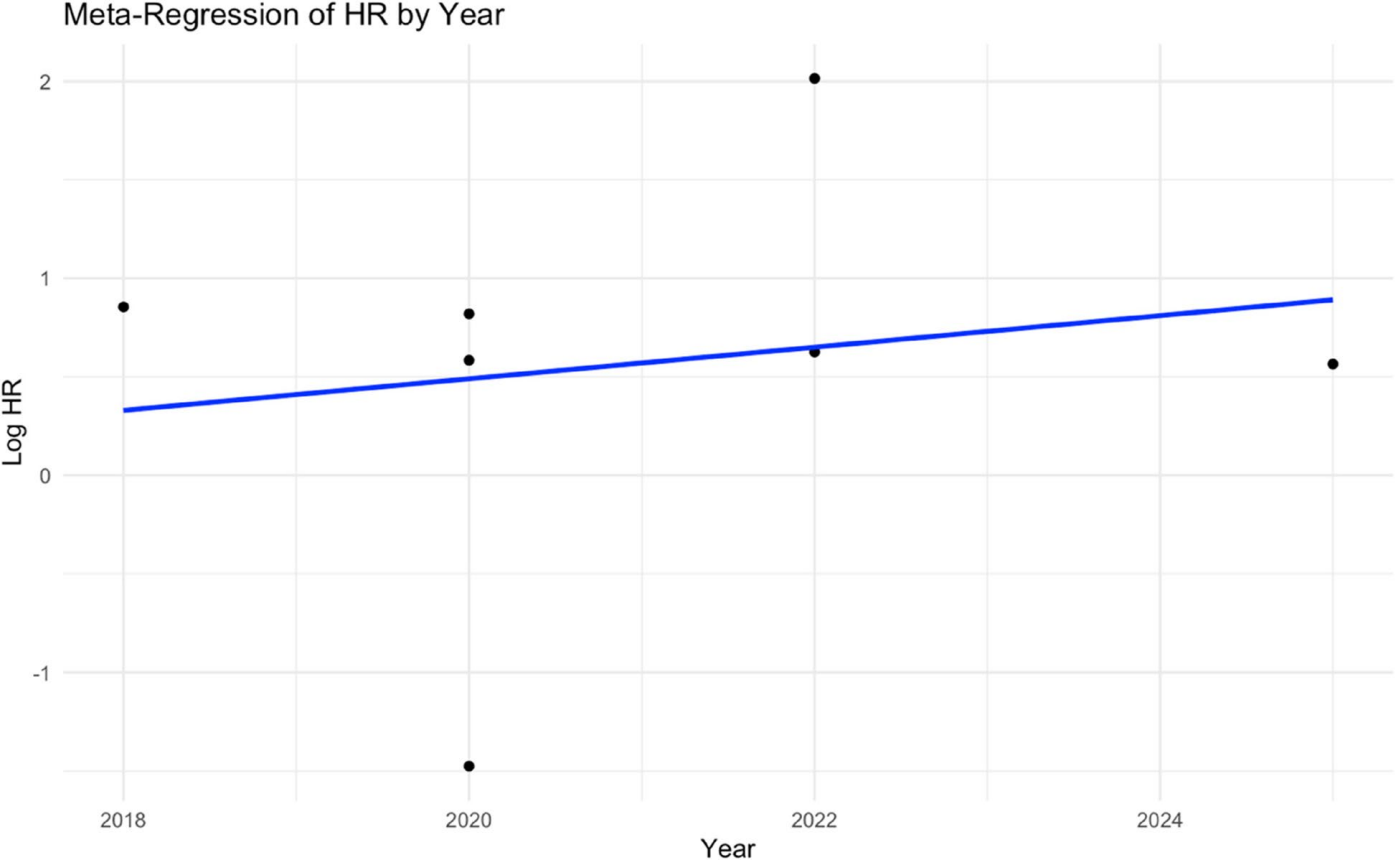
Meta-regression of publication year and log HR in gastric cancer patients

A meta-regression analysis demonstrated a positive trend between publication year and log HR, indicating an increase in the estimated effect size over time, although the association was not statistically significant (*p* > 0.05) (Fig. 5).

Meta-regression analyses using mixed-effects models with restricted maximum likelihood (REML) did not identify mean age or sex distribution as significant moderators of the prognostic effect of PD-L1 expression (Table 3).

**Table 3:** Meta-regression analyses of study-level moderators influencing the prognostic value of PD-L1 expression (mixed-effects model, REML)

Moderator	β (log HR)	p-value
Mean age (years)	0.0388	0.353
Male proportion (%)	− 0.0030	0.954

### Certainty of evidence assessment

Table 4 presents the certainty of evidence assessment using the GRADE framework. According to the assessment results, the certainty of evidence was rated as moderate, as no serious concerns were noted regarding risk of bias, inconsistency, indirectness, imprecision, or publication bias.

**Table 4:** Certainty of evidence assessment

Outcome	No. of studies	Study design	Risk of bias	Inconsistency	Indirectness	Imprecision	Publication bias	Effect estimate HR	Certainty of evidence
Overall survival (HR)	7	Observational studies	Not serious	Not serious	Not serious	Not serious	Undetected	1.70 (1.13–2.55)	Moderate

## Discussion

This meta-analysis demonstrated a statistically significant association between PD-L1 expression and overall survival in patients with GC. However, the pooled hazard ratio alone does not fully explain the biological mechanisms underlying the observed prognostic associations. Therefore, interpretation of these findings requires consideration of the complex interactions between tumor cells and the tumor immune microenvironment, particularly the regulatory pathways involved in immune checkpoint signaling.

In the present systematic review and meta-analysis, we evaluated the prognostic significance of PD-L1 expression in GC using evidence published between 2018 and 2025. Although PD-L1 is widely recognized as a predictive biomarker for response to immune checkpoint inhibitors in advanced GC, its independent prognostic value remains controversial. This uncertainty is also reflected in current clinical guidelines and recent comprehensive reviews, which emphasize the heterogeneity of PD-L1–related outcomes and caution against interpreting PD-L1 expression as an isolated prognostic factor in routine clinical practice [33–35].

Our pooled analysis demonstrated a significant association between PD-L1 expression and overall survival in GC patients, although considerable between-study heterogeneity was observed. Importantly, the prognostic association appeared to be influenced by the methodological approach used for PD-L1 assessment. Studies employing the combined positive score (CPS ≥ 1) showed more consistent prognostic associations compared with those using semi-quantitative scoring systems. These findings are consistent with several recent cohort studies. For example, in a study of GC patients with peritoneal metastasis, high PD-L1 expression (CPS ≥ 10) was identified as an independent predictor of longer overall survival, which may reflect the presence of an activated anti-tumor immune response in this patient subgroup [36]. Similarly, a study conducted in a Jordanian population reported that PD-L1 positivity (CPS ≥ 1) was associated with improved survival outcomes [37].

Nevertheless, the literature also contains studies demonstrating an unfavorable prognostic impact of PD-L1 expression in GC. For example, Dung et al. (2022) reported that PD-L1 positivity defined by CPS ≥ 1 was significantly associated with poorer overall survival in GC patients [38]. Comparable findings were reported in an umbrella meta-analysis, where PD-L1 positivity was associated with poorer survival outcomes [39]. Similarly, the meta-analysis by Qiu and Du (2021) demonstrated that positive PD-L1 expression was associated with poorer overall survival and adverse clinicopathological characteristics, including lymph node metastasis, although considerable heterogeneity across studies was noted [40]. In addition, retrospective cohort studies conducted in specific populations, such as GC patients in Thailand, also reported worse survival outcomes among PD-L1–positive patients [41]. Taken together, these findings suggest that the prognostic role of PD-L1 in GC may be context-dependent.

Recent evidence increasingly indicates that the prognostic significance of PD-L1 expression in GC is closely related to the composition and functional state of the tumor immune microenvironment. In surgically treated GC patients, particularly those with stage I–III disease, PD-L1 expression may reflect an active anti-tumor immune response characterized by infiltration of tumor-infiltrating lymphocytes. In such cases, PD-L1 upregulation may represent an adaptive immune resistance mechanism induced by interferon-γ signaling in response to cytotoxic T-cell activity. Consequently, PD-L1 positivity may be associated with enhanced immune surveillance and more favorable clinical outcomes in certain biological contexts [42, 43].

This immune-active microenvironment has been associated with improved survival outcomes and increased responsiveness to immunotherapy, particularly in molecular subtypes characterized by microsatellite instability (MSI-high) or Epstein–Barr virus (EBV) positivity [44, 45]. Conversely, in immunologically “cold” tumors with limited effector immune cell infiltration or dominant immunosuppressive components, such as regulatory T cells or myeloid-derived suppressor cells, PD-L1 expression may reflect tumor immune evasion rather than effective anti-tumor immunity. This may explain the unfavorable prognostic associations observed in some studies [46, 47].

Our meta-regression analysis demonstrated no significant modifying effects of publication year, mean age, or sex distribution on the prognostic value of PD-L1 expression in GC. These findings are consistent with several previous studies and systematic reviews indicating that the association between PD-L1 expression and survival outcomes is largely independent of demographic factors such as age and sex [48, 49]. Indeed, multiple cohort studies have reported comparable prognostic effects of PD-L1 expression across different age groups, suggesting that immunological aging does not substantially alter its prognostic relevance in GC [50].

Similarly, although sex-related differences in immune responses and tumor microenvironment composition have been described, current evidence does not support a consistent sex-specific impact of PD-L1 expression on overall or disease-free survival in GC patients [51–53]. Taken together, these observations suggest that intrinsic tumor-related biological characteristics—including immune microenvironment composition, molecular subtype, and genomic instability—are more likely to determine the prognostic value of PD-L1 expression than patient-related demographic factors. For this reason, the prognostic significance of PD-L1 expression in GC should be interpreted within the context of tumor immune microenvironment characteristics and methodological differences in PD-L1 assessment.

### Limitations

Several limitations of this study should be acknowledged. First, substantial between-study heterogeneity was observed, which may reflect differences in PD-L1 assessment methods (CPS vs. semi-quantitative scoring), cut-off values, patient populations, disease stages, as well as variability or incomplete reporting of antibody clones used for PD-L1 immunohistochemical detection. These methodological differences limit the comparability of individual studies and may contribute to heterogeneity in the pooled estimates.

Second, the overall certainty of evidence can be considered moderate, as most included studies were retrospective and therefore subject to residual confounding and potential selection bias.

Third, the limited availability of individual patient-level data precluded more detailed subgroup analyses according to molecular tumor subtypes or treatment modalities. In addition, due to the limited number of included studies (*n* = 7), further meta-regression analyses incorporating additional moderators, such as tumor stage, PD-L1 positivity rate, and antibody clone, were not statistically feasible.

## Conclusion

In conclusion, the present systematic review and meta-analysis suggest that PD-L1 expression is associated with overall survival in patients with GC, although the direction and magnitude of this association appear to be context-dependent.

The prognostic effect was more consistent in studies employing the CPS, underscoring the importance of standardized PD-L1 scoring methods in GC research. These findings emphasize that PD-L1 expression should be interpreted in conjunction with tumor immune microenvironment characteristics and molecular tumor subtypes in order to improve prognostic stratification in GC.

### Future research directions

Future research should focus on large multicenter prospective cohort studies designed to standardize PD-L1 detection methods, including antibody clone selection and scoring criteria. In addition, future investigations should integrate PD-L1 assessment with molecular subtypes of GC, particularly MSI and EBV status, in order to better clarify the biological and prognostic significance of PD-L1 expression.

## Data Availability

The data used and/or analyzed during the current study are available from the corresponding author on reasonable request.
